# Efficiency as a determinant of loyalty among users of a Community of Clinical Practice: a comparative study between the implementation and consolidation phases

**DOI:** 10.1186/s12875-020-1081-x

**Published:** 2020-01-24

**Authors:** David Lacasta Tintorer, Josep Maria Manresa Domínguez, Ana Jiménez-Zarco, Teresa Rodríguez-Blanco, Souhel Flayeh Beneyto, Pere Torán-Monserrat, Xavier Mundet Tuduri, Francesc Saigí-Rubió

**Affiliations:** 1 Centre d’Atenció Primària Gran Sol, Gerència d’Àmbit d’Atenció Primària Metropolitana Nord, Institut Català de la Salut. Avinguda del Doctor Bassols, 112 - 130, 08914 Badalona, Spain; 2 Unitat de Suport a la Recerca Metropolitana Nord, IDIAP Jordi Gol, CAP El Maresme. Camí del Mig, 36 planta 4ª, 08303 Mataró, Spain; 3 Universitat Autònoma de Barcelona, Bellaterra, Cerdanyola del Vallès. Campus de la UAB, Plaça Cívica, s/n, 08193 Bellaterra, Barcelona, Spain; 4 Faculty of Economics and Business, Universitat Oberta de Catalunya, Barcelona, Spain; 5 Institut Universitari d’Investigació en Atenció Primària (IDIAP Jordi Gol), Gran Via Corts Catalanes, 587, àtic, 08007 Barcelona, Spain; 6 Departament de Ciències Mèdiques, Universitat de Girona, C/ Emili Grahit, 77, 2n, 17003 Girona, Spain; 7 Unitat de Suport a la Recerca Barcelona Ciutat, IDIAP Jordi Gol, Carrer Sardenya 375, 08025 Barcelona, Spain; 8 Faculty of Health Sciences, Universitat Oberta de Catalunya, Barcelona. Av. Tibidabo, 39-43, 08035 Barcelona, Spain

**Keywords:** Remote consultation, Primary health care, Problem solving, Telemedicine, Referral and consultation, Continuing medical education

## Abstract

**Background:**

A community of clinical practice called the *Online Communication Tool between Primary and Hospital Care* (ECOPIH) was created to enable primary care and specialist care professionals to communicate with each other in order to resolve real clinical cases, thereby improving communication and coordination between care levels. The present work seeks to analyse whether ECOPIH makes it possible to reduce the number of referrals. To that end, the objectives are: (1) To find out the degree of loyalty among ECOPIH users, by comparing the medical professionals’ profiles in the tool’s implementation phase to those in its consolidation phase. (2) To evaluate the degree of fulfilment of users’ expectations, by establishing the determining factors that had an influence on the physicians’ intention to use ECOPIH in the implementation phase and observing whether its use had an effective, direct impact on the number of patient referrals that primary care physicians made to specialist care professionals.

**Methods:**

Two studies were conducted. Based on a survey of all the physicians in a Primary Care area, Study 1 was a descriptive study in ECOPIH’s implementation phase. Study 2 was a randomised intervention study of ECOPIH users in the tool’s consolidation phase. The results from both studies were compared. Various bivariate and multivariate statistical techniques (exploratory factor analysis, cluster analysis, logistic regression analysis and ANOVA) were used in both studies, which were conducted on a sample of 111 and 178 physicians, respectively.

**Results:**

We confirmed the existence of an ECOPIH user profile stable across both phases: under-50-year-old women. Regarding the second objective, there were two particular findings. First, the discriminant factors that had an influence on greater ECOPIH use were habitual *Social media website and app use* and *Perceived usefulness for reducing costs*. Second, PC professionals who were ECOPIH members made fewer referrals to SC professionals in Cardiology, Endocrinology and Gastroenterology than older PC professionals who were not ECOPIH members.

**Conclusions:**

The use of a community of clinical practice by primary care and specialist care professionals helps to reduce the number of referrals among medical professionals.

## Background

In the current context of healthcare spending containment, the role of primary care (PC) is fundamental because, when managed effectively, it can prevent unnecessary referrals and reduce waiting lists [1–3]. However, people with multiple and complex health problems are cared for in PC clinics [4]. This means that physicians have to deal with several clinical aspects of patients at once; physicians may have doubts about how to manage complex patient needs in day-to-day clinical practice [5–8]. PC professionals therefore need an effective system that allows them to perform searches and find the necessary information to enhance their knowledge and find suitable solutions [9].

Face-to-face or telephone discussions with specialist care (SC) professionals enable PC professionals to address particular clinical concerns that crop up during patient care. However, given that the health system is at saturation point, communication between PC and SC may be difficult, slow, and ineffective [10–13], and it leads to many referrals to SC (hospitalisation or specialist outpatient clinics). In turn, this leads to excessive delays for appointments [14, 15] and to a significant increase in financial, time and psychological costs to physicians and patients. As Horner et al., have pointed out, 65% of referrals are inappropriate and up to 30% of them could be avoided [16].

Among the factors associated with a higher referral rate are the little coordination between care levels and the lack of training [10, 17–19]. Improving coordination between care levels would not only enhance healthcare, but also be of considerable educational value and lead to a more cost-effective use of health services [2, 20]. Telemedicine can improve communication between PC and SC, and thereby improve efficiency, cost-effectiveness, and medical care quality [21–27], with a high degree of patient satisfaction [28–30].. In addition, telemedicine can reduce the number of supplementary tests and referrals to SC (by between 8.9 and 51%) [30–32].

The formation of communities of practice (CoPs) is a recent approach [33]. Applied to the field of healthcare, communities of clinical practice (CoCPs) are online platforms that draw on the advantages of Web 2.0 to construct knowledge among healthcare professionals working at different levels of care [34]. Although there is limited evidence of their usefulness [35, 36], CoCPs have been shown to have considerable capacity to enable the transfer of knowledge gained in day-to-day practice [37–39], as well as a lot of potential in terms of professionals’ education, regardless of their care level [36, 40–43].

Set up in 2009, *Eina de Comunicació Online entre Primària i Hospitalària* (ECOPIH as abbreviated in Catalan, or Online Communication Tool between Primary and Hospital Care as translated in English) is a CoCP based on a Web 2.0 platform. It facilitates communication between PC and SC professionals respectively working at a number of PC centres and hospitals in the cities of Badalona and Sant Adrià de Besòs in greater Barcelona, Spain [44]. It enables PC and SC professionals to share up-to-date information that is relevant to their interests, and PC professionals to raise clinical cases for consultation with specialists to improve patient management and to reduce the number of referrals to the next care level. After a two-year follow-up period (2011–2012), 1000 interventions had been made across six specialities through ECOPIH. Contributions had been read 12,200 times (each contribution approximately 10 times) and 209 clinical cases had been raised for consultation.

**Fig. 1:**
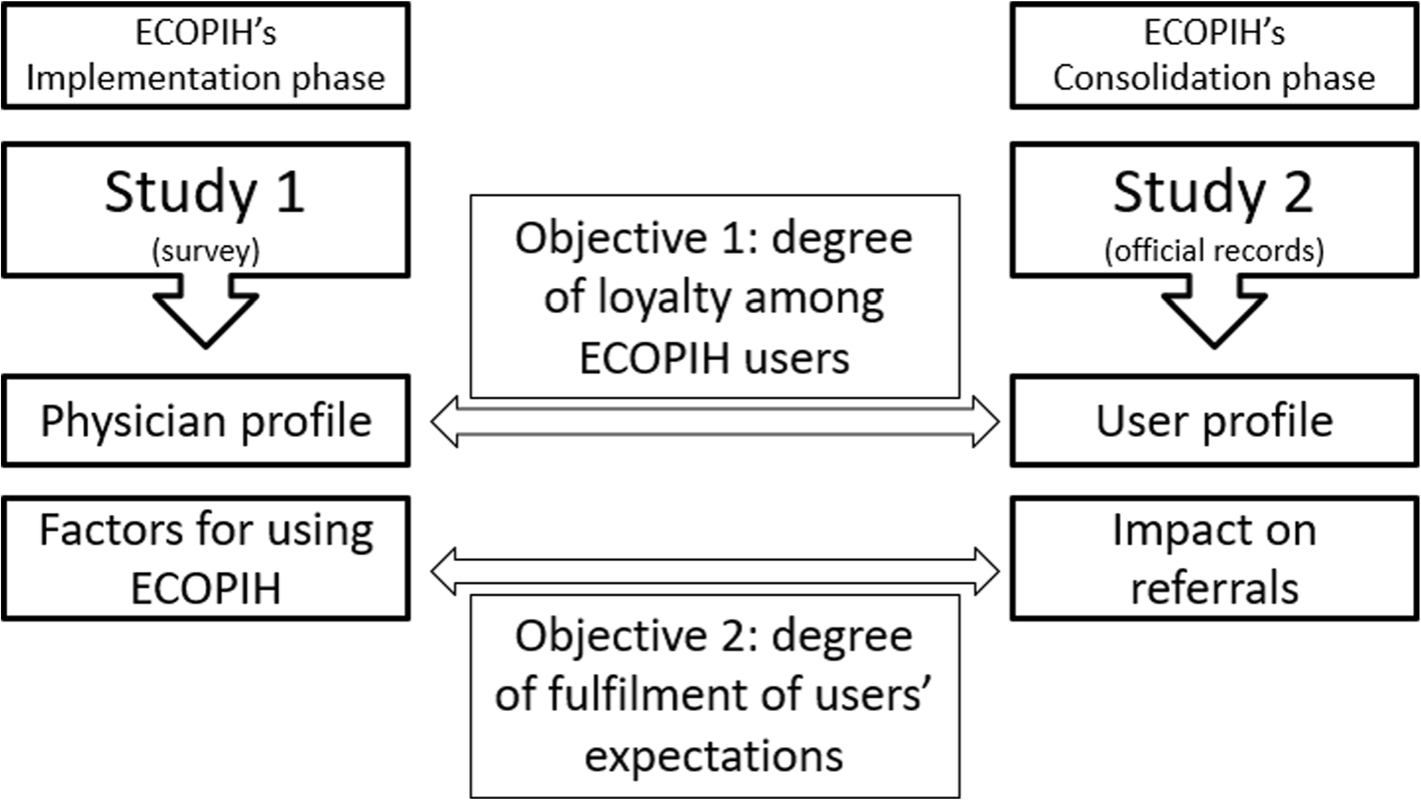
Objectives and results obtained from the two studies conducted in the present work

Presented in this article are the results from two ECOPIH follow-up studies conducted between 2011 and 2012, coinciding with the respective implementation (first year of ECOPIH use) and consolidation phases of that CoCP (end of the second year of use). Study 1 evaluated the discriminant factors that had an influence on the intention to use ECOPIH, and Study 2 performed a characterisation of ECOPIH users and analysed the impact of ECOPIH use on referrals. By comparing the results obtained from the two studies conducted, the present work seeks to analyse whether ECOPIH makes it possible to reduce the number of referrals to SC. To that end, the objectives are (Fig. 1):

(1) To find out the degree of loyalty among ECOPIH users. Specifically, by identifying and then comparing the medical professionals’ profiles in the tool’s initial implementation phase (Study 1) to those in its consolidation phase (Study 2) to see if they remained stable.

(2) To evaluate the degree to which users’ expectations are fulfilled by establishing the determining factors that influence physicians’ intention to use ECOPIH in the implementation phase (Study 1) and analysing whether the expectations identified in that phase were fulfilled by observing whether its use had an effective, direct impact on the number of patient referrals that PC physicians made to SC professionals (Study 2).

## Methods

The research presented in this article is the result of a collaboration between the Badalona-Sant Adrià de Besòs Primary Care Service (PCS) in Catalonia, Spain, and the Open University of Catalonia (UOC, as abbreviated in the Catalan language), Spain. Some of the results obtained from this study relating to physicians’ and nurses’ adoption of the tool have been published elsewhere [45].

The Badalona-Sant Adrià de Besòs PCS includes nine PC centres and three SC centres: Germans Trias i Pujol University Hospital, Badalona Municipal Hospital and the Barcelonès Nord International Health Unit, with a total of 624 healthcare professionals. These centres serve 227,151 inhabitants.

Two studies were conducted. Study 1 was a descriptive study in ECOPIH’s implementation phase. Study 2 was a randomised intervention study of ECOPIH users conducted two years later, when use of the tool had become consolidated.

### Study 1 (implementation phase): characterisation of ECOPIH users during the implementation phase and description of discriminant factors that have an influence on the intention to use ECOPIH

#### Settings, sampling and type of study

Study 1 was a descriptive study on a sample of healthcare professionals from the Badalona-Sant Adrià de Besòs PCS, who had the potential to become regular users of ECOPIH. An anonymous, optional survey consisting of open and closed questions was used to gather data [45].

The questionnaires were divided into three sections: 1) sociodemographic and professional background; b) access to and use of Information and Communication Technologies (ICTs) in professional and personal settings; and c) perceptions and use of ECOPIH. Information about the scientific objectives and data confidentiality was made available to potential respondents. A total of 357 healthcare professionals were invited, by e-mail, to fill in the questionnaire. All of those professionals were (a) involved in caring for patients in the Badalona-Sant Adrià de Besòs PCS and (b) could potentially become routine ECOPIH users. All professionals were invited to take part by e-mail, though it was made clear that they could decline if they wished. The questionnaires were provided in additional files 1 and 2. The study sample was formed by a total of 111 physicians who filled in the questionnaire (31.9% response rate). The study was conducted over a two-month period, from 1 December 2011 to 31 January 2012. As reflected by the above-mentioned percentage, the high response rate meant that it was a representative sample of the ECOPIH community population. Also, given the population’s finite size, it was possible to work with low margins of error (+ 7.7, 95% confidence level). This meant that the results could be extrapolated. Table 1 presents the study specifications, and Table 2 the variables used in the study.

**Table 1:** Study specifications

	Study 1	Study 2
Universe	357 healthcare professionals	357 physicians
Sample	111 physicians	178 physicians
Margin of error	7.7% (p = q) 95% confidence level	5.2% (*p* = q) 95% confidence level
Data collection method	Questionnaire	Official or institutional electronic records, from the clinical records management program, 2010 to 2012
Sampling method	Random^a^	Random^b^
Background work	December 2011	December 2012

^a^Based on an anonymous survey.

^b^Randomised intervention study of ECOPIH users.

#### Variables of study 1

The use of ECOPIH is a dependent variable and the rest are independent (explanatory) ones (Table 2). It should be pointed out that principal component analysis was used to obtain the two variables measuring perceived usefulness – *Perceived usefulness for improving clinical practice quality*, and *Perceived usefulness for reducing costs*. It was the multidimensional nature of these variables that suggested exploratory factor analysis (EFA) should be performed. EFA is a technique to reduce data dimensionality. By analysing a set of original variables, it seeks to determine the fewest dimensions capable of explaining the maximum amount of information within the data [46].

**Table 2:** Variables used in Study 1

Model variable		
The use of ECOPIH		The healthcare professional uses ECOPIH. Dichotomous variable, where 0 = no and 1 = yes.
Perceived usefulness of ECOPIH	Perceived usefulness for improving clinical practice quality (PU1)	Metric variable obtained from a principal component analysis (see Annex 2) determining the extent to which the healthcare professionals perceived that ECOPIH use improved clinical practice quality.
	Perceived usefulness for reducing costs (PU2)	Metric variable obtained from a principal component analysis (see Annex 2) determining the extent to which the healthcare professionals perceived that ECOPIH use reduced clinical practice costs (in time and effort invested in getting hold of information).
Perceived ease of use of ECOPIH		Variable measured on a 5-point Likert scale indicating the healthcare professionals’ perceived ease of use of ECOPIH.
Security and confidentiality		Variable measured on a 5-point Likert scale indicating the level of patient data security and confidentiality that ECOPIH has.
Healthcare professional profile		Dichotomous variable indicating the individual’s professional profile. 1 = physician and 0 = nurse.
ICT user profile	Mobile device use	Categorical variable indicating the extent to which the ICT user uses different types of mobile device. 1 = low, 2 = medium, 3 = high, 4 = very high.
	Social media website and app use	Categorical variable indicating the extent to which the ICT user uses social media technologies (access to social networks). 1 = low, 2 = medium, 3 = high and 4 = very high.
Gender		Gender of the healthcare professional. 1 = female and 0 = male.
Age		Age of the healthcare professionals. The variable has four values: 1 = under 40 years old, 2 = between 40 and 49 years old, 3 = between 50 and 59 years old, and 4 = 60 years old or over.

Source: Lacasta et al. [45]

In total, nine variables were considered for the purpose of extracting the factor dimensions. Each variable was associated with the healthcare professionals’ perceived benefits of using ECOPIH. Regarding the particular benefits that ECOPIH could offer its users, some of these variables referred to quality improvement, while others referred to cost reduction (see Table 3 in Lacasta et al. [45]).

As the 2015 study showed [45], performing a set of statistical tests enabled us to establish the suitability of the analysis and the reliability of the scale. All of the correlation matrix’s variables displayed high correlations, and the value of their determinant was 0.041. The Kaiser-Meyer-Olkin index value was 0.924 and Bartlett’s test of sphericity value was 1983.717, with a significance of 0.000. This analysis explained 86.846% of the variance, and Cronbach’s alpha values were higher than 0.81 in all the scales. According to Nunnally [47], this indicator must have values higher than 0.7 in general and higher than 0.6 in the case of new scales. Thus, it is possible to assume that the scales used were reliable. In addition, the discriminant, convergent and nomological validity of the content and construct scales was addressed. Regarding the content, the scales were developed following a major review of the literature (see Table 3 in Lacasta et al. [45]).

In order to establish the physicians’ profiles, univariate analyses were performed on the different sociodemographic and ICT use variables for the selected sample. An important matter was to identify the physicians’ profiles by gender and age. To that end, hierarchical cluster analysis was carried out.

To identify the variables determining ECOPIH use, binary logistic regression analysis (Logit) was performed.

### Study 2 (consolidation phase): characterisation of ECOPIH users during the consolidation phase and impact of ECOPIH use on referrals

#### Settings, sampling and type of study

Study 2 was an open, multi-centre, controlled, randomised intervention study over a 24-month follow-up period. It was conducted on three PCSs in Barcelona Province (Badalona-Sant Adrià de Besòs PCS, SAP Santa Coloma de Gramenet PCS and Maresme PCS), with 25 PC centres and 507 PC physicians, all belonging to the public health system of Catalonia, Spain.

The inclusion criteria were: PC clinicians who had been working for at least 6 months at the same PC centre for whom full patient visit and referral data were available in the official electronic records of the institution. Since only adult medicine specialities were analysed, PC paediatricians were excluded.

The population of 357 physicians at the Badalona-Sant Adrià de Besòs, Santa Coloma de Gramenet and Maresme PCSs was the reference point, from which a sample comprising 178 physicians was taken. It should be noted that this random sample was different from the one used in Study 1, although the study universe was the same – and finite – in both studies.

#### Variables of study 2

In order to establish the healthcare professionals’ profiles, hierarchical cluster analysis was performed, taking into account the *Gender*, *Age* and *ECOPIH member* variables.

The Background work for Study 2 was carried out in December 2012. Table 3 shows the variables analysed in that study.

**Table 3:** Variables used in Study 2

Model variable	
Ecopih member	The healthcare professional is a member of ECOPIH. Dichotomous variable. 0 = no and 1 = yes.
Gender	Gender of the healthcare professional. 1 = female and 0 = male.
Age	Age of the healthcare professional. The variable has four values: 1 = under 40 years old, 2 = between 40 and 49 years old, 3 = between 50 and 59 years old, and 4 = 60 years old or over.
Referrals made	Dependent variable. The number of patient referrals that the PC professional made in the last year. Categorical variable. 1 = low referral rate (fewer than 7 referrals), 2 = average referral rate (between 7 and 14 referrals), 3 = high referral rate (between 14 and 23 referrals) and 4 = very high referral rate (more than 23 referrals).

In order to respond to the first objective, the healthcare professionals’ profiles were defined according to the intention to use the CoCP in the implementation phase (Study 1). After the tool’s consolidation, the professionals’ profiles were analysed again, taking into account the effective use of the tool (Study 2). By comparing the profiles obtained from the two studies, it was possible to find out if potential users had become actual users (loyalty).

Regarding the second objective, in order to confirm whether the tool actually had an influence on the professionals’ behaviour, an analysis was performed of the relationship between the profiles of the professionals using ECOPIH (Study 1) and the number of referrals they made in certain specialities (Study 2). Chi-square analysis was used to analyse the relationship of dependence between the variables.

### Ethics approval

This project adhered to Spanish legislation (Spanish Law 14/2007 of 3 July on Biomedical Research) and to international regulations on ethical issues (Declaration of Helsinki and Declaration of Tokyo). The research protocol (P11/39) was reviewed and approved by the Ethics and Clinical Research Committee of the Primary Care Research Institute IDIAP Jordi Gol, Barcelona, Spain. All the participants were informed in writing about their participation in the study and data confidentiality. In order to avoid bias, no information about the intervention was provided. Written informed consent was obtained from all participants. Confidentiality was maintained at all levels, thereby ensuring that professionals and patients could not be identified. The patients’ medical records could not be accessed from ECOPIH. Information was obtained from the survey responses and existing data related to visits and referrals, and subject-identifying information was coded and anonymised. The features of the intervention meant that it did not have to meet national regulations for clinical trials. Confidentiality was assured under the Spanish Personal Data Protection Law (15/1999 of 13 December).

## Results

### First objective: to find out the degree of loyalty among ECOPIH users

#### Implementation phase: sample profile study 1

At the start of ECOPIH implementation, sample distribution by sex and age was fairly balanced: 56.9% women and 43.1% men. Regarding age, 32.4% were under 40 years old, 25.2% were between 40 and 49 years old, 29.7% were between 50 and 59 years old, and only 12.6% were 60 years old or over. Finally, it should be noted that the large majority’s *Mobile device use* was medium-high (70.7%), whereas their *Social media website and app use* was medium (59.8%) or low (38.3%) (Table 4).

**Table 4:** Descriptive statistics of the sample (Study 1)

		physicians (111)	Profile 1A (64)	Profile 1B (47)
gender	Female	63 (56.9%)	42 (65.6%)	22 (46.8%)
	Male	48 (43.1%)	22 (34.4%)	25 (53.2%)
age	< 40 years old	36 (32.4%)	36 (56.2%)	0
	40–49 years old	28 (25.2%)	28 (43.8%)	0
	50–59 years old	33 (29.7%)	0	33(70.2%)
	≥ 60 years old	14 (12.6%)	0	14 (29.8%)
Mobile device use	Low	24 (22.0%)	11 (17.2%)	13 (27.7%)
	Medium	39 (34.9%)	21 (32.8%)	17 (36.2%)
	High	40 (35.8%)	27 (42.2%)	14 (29.4%)
	Very high	8 (7.3%)	5 (7.8%)	3 (6.4%)
Social media website and app use	Low	43 (38.3%)	26 (41.3%)	31 (65.2%)
	Medium	66 (59.8%)	36 (56.6%)	16 (34.8%)
	High	2 (1.9%)	2 (3.2%)	0
Intention to use ecopih	Yes	59 (53.2%)	35 (54.7%)	24 (51.1%)
	No	52 (46.8%)	29 (45.3%)	23 (48.9%)

As shown in Table 5, the results obtained indicated the existence of two different groups. The first profile (1A) comprised 64 individuals, of whom 65.6% were under-50-year-old women, and the second profile (1B) comprised 47 individuals, of whom 53.2% were over-50-year-old men. The differences between the two groups were significant for both the *Age* variable and the *Gender* variable, with t-test values of 8.708 and 4.437, respectively, at 99 and 95% confidence levels (Table 5).

**Table 5:** Main cluster results at the start of ECOPIH implementation

		Profile 1A	Profile 1B	Significance (t-test)
Age	< 40 years old	62 (56.2%)	0	8.708 (0.004)
	40–49 years old	49 (43.8%)	0	
	50–59 years old	0	78 (70.2%)	
	≥ 60 years old	0	33 (29.8%)	
Gender	Male	38 (34.4%)	59 (53.2%)	4.437 (0.032)
	Female	73 (65.6%)	52 (46.8%)	
Final cluster centres				
Gender		1	2	
Age		1.44	3.30	

When analysing each group’s relationship with technology, the distribution with respect to *Social media website and app use* was found to be quite homogenous and similar in both, hence the Chi-square value was not significant in either of them.

#### Consolidation phase: analysis of the ECOPIH users’ profiles. Sample profile study 2

As shown in Table 6, the results obtained indicated the existence of two different groups. The first profile (2A) comprised 72 professionals under 50 years old (100%), most of whom were women (76.4%) and ECOPIH members (68.1%), and the second group (Profile 2B) comprised 106 individuals over 50 years old (100%), most of whom were women (62.3%) and not ECOPIH members (85.8%).

**Table 6:** Main cluster results (Study 2)

		Profile 2A (72)	Profile 2B (106)	Significance (t-test)
Age	< 40 years old	32 (44.4%)	0	43.250 (0.000)
	40–49 years old	40 (55.6%)	0	
	50–59 years old	0	86 (81.1%)	
	≥ 60 years old	0	20 (18.9%)	
Gender	Male	17 (23.6%)	40 (37.7%)	
	Female	55 (76.4%)	66 (62.3%)	
ECOPIH member	Yes	49 (68.1%)	15 (14.2%)	
	No	23 (31.9%)	91 (85.8%)	
Final cluster centres				
Gender		2	2	
Age		1.56	3.19	
ECOPIH member		1	9	

The healthcare professionals’ profiles obtained from the analysis of the sample in the first year (Study 1, implementation phase) coincided with those obtained in the second year of ECOPIH development (Study 2, consolidation phase). Thus, a professional profile was observed in both the implementation and the consolidation phases (profiles 1A and 2A) corresponding to healthcare professionals who were young, mostly women, and habitual users of technology. In another profile (profiles 1B and 2B in the respective stages), the healthcare professionals were older, mostly men, whose ICT use was lower. This explains why the first segment’s use of ECOPIH (profile 1A and 2A) was high (68.1%) and the second group’s use of the tool (profile 1B and 2B) was very low (14.2%). We can therefore confirm the existence of an ECOPIH user profile – under-50-year-old women who habitually use ICTs – that was stable in both the implementation phase and the consolidation phase, thus maintaining their loyalty to the tool.

### Second objective: to evaluate the degree of fulfilment of users’ expectations

#### Implementation phase: determinants of ECOPIH use

The model’s goodness of fit was confirmed by the values and level of significance of the Chi-square statistic (68.228, sig. 0.000) and the Hosmer-Lemeshow test (10.224, *p* = 0.250). In addition, the value of Nagelkerke’s statistic indicated that the model obtained explained 62.1% of the dependent variable’s variance.

From the analysis in Table 7, it is possible to observe that the variables influencing frequency of use are, on the one hand, the user’s profile in terms of his or her frequency of *Social media website and app use* (B = 1.933 *p* = 0.002) and, on the other, *Perceived usefulness for reducing costs* (time and financial costs) that ECOPIH use entails (B = 1.706 *p* = 0.025). No significant differences were found taking into account the professionals’ gender or age.

**Table 7:** Equation variables (Study 1)

	B	E.T.	Wald	DF	Sig.	Exp(B)
Perceived usefulness for reducing costs (PU2)	1.706	0.761	5.026	1	0.025	5.508
Perceived usefulness for improving clinical practice quality (PU1)	0.793	0.636	1.553	1	0.213	2.211
Perceived ease of use of ECOPIH	−0.075	0.310	0.058	1	0.810	0.928
Security and confidentiality	0.016	0.311	0.003	1	0.958	1.016
Social media website and app use	1.933	0.619	9.748	1	0.002	6.907
Mobile device use	−0.011	0.339	0.001	1	0.973	0.989
Constant	−3.327	1.751	3.612	1	0.057	0.036

#### Consolidation phase: impact of ECOPIH on referrals

Table 8 shows that the PC professionals’ behaviour was significantly different for three of the specialities analysed. Thus, it was found that, ECOPIH members in PC professions made a low or average number of referrals to SC professionals in Cardiology, Endocrinology, and Gastroenterology, whereas older professionals who were not members of ECOPIH made a high or very high number of referrals. No significant differences were found with regard to the number of referrals made by each group at the start of the study period, so the differences found could be related to ECOPIH use.

**Table 8:** Referrals in different specialities, by professional profile (Study 2)

Referral rate by speciality		Profile 2A (women, < 50 years old, ECOPIH users)	Profile 2B (men, > 50 years old, non-ECOPIH users)	SIGNIFICANCE (*F* value)
Cardiology	Low	11 (15.3%)	5 (4.7%)	0.023
	Average	7 (9.7%)	6 (5.7%)	
	High	18 (25.0%)	21 (19.8%)	
	Very high	36 (50.0%)	74 (69.8%)	
Endocrinology	Low	18 (25.0%)	10 (9.4%)	0.025
	Average	12 (16.7%)	29 (27.4%)	
	High	21 (29.2%)	29 (27.4%)	
	Very high	21 (29.2%)	38 (36.8%)	
Gastroenterology	Low	9 (12.5%)	5 (4.7%)	0.019
	Average	9 (12.5%)	7 (6.6%)	
	High	17 (23.6%)	16 (15.1%)	
	Very high	37 (51.4%)	78 (73.6%)	
Nephrology	Low	66 (91.7%)	94 (88.7%)	0.635
	Average	6 (8.3%)	11 (10.4%)	
	High	0 (0.0%)	1 (0.09%)	
	Very high	–	–	
Neurology	Low	12 (16.7%)	10 (9.4%)	0.455
	Average	9 (12.5%)	13 (12.3%)	
	High	19 (26.4%)	36 (34.0%)	
	Very high	32 (44.4%)	47 (44.3%)	
Respiratory Medicine	Low	11 (15.3%)	11 (10.4%)	0.443
	Average	15 (20.8%)	17 (16.0%)	
	High	17 (23.6%)	35 (33.0%)	
	Very high	29 (40.3%)	43 (40.6%)	

For the remaining specialities (Nephrology, Respiratory Medicine and Neurology), no differences were found in the number of referrals. However, it should be noted that the three specialities in which differences were found were those that involved higher total numbers of referrals and healthcare professionals.

## Discussion

The analysis of the results of the two studies shown has enabled us to respond to the two stated objectives:

(1) To find out the degree of loyalty among ECOPIH users, and;

(2) To evaluate the degree of fulfilment of users’ expectations.

Both objectives are closely related since one of the main reasons why potential users become actual users is the fulfilment of their expectations from the tool.

Regarding the first objective, after comparing the professional profiles in the implementation phase to those in the consolidation phase, two professional profiles were found to remain similar over time. In one profile, the healthcare professionals were young, mostly women, and habitual users of technology. In another, the healthcare professionals were older, mostly men, whose ICT use was lower.

The findings suggest that the degree of loyalty (from initial use to consolidated use) was high among the group of younger female professionals. This seems to be supported by the fact that those professionals in the segment comprising mostly under-50-year-old women who stated their intention to use ECOPIH in the implementation phase were actual members of it two years later.

The results obtained show that the professionals in the over-50-year-old profile were those who used ECOPIH to a lesser extent, as identified in Study 2. This segment bears considerable similarity to Profile 1B observed in Study 1 (except for gender), in which the professionals rated information security higher than cost reduction.

In order to respond to the second objective, the determining factors that had an influence on the use of ECOPIH in the implementation year were analysed. The impact of the tool’s use on the number of referrals in an uncontrolled real-life setting was then evaluated, based on the medical professionals’ voluntary use thereof. This enabled us to establish whether the expectations created at the start of ECOPIH use (specifically, that PC professionals felt that ECOPIH use would allow costs associated with clinical practice to be reduced), had been fulfilled.

Concerning the factors determining the adoption of ECOPIH, our study revealed that two factors explained physicians’ use of this tool. Firstly, professionals’ ICT user profiles influenced intention to use ECOPIH (B = 1.933 *p* = 0.002). All professionals, regardless of their age and whether or not they were ECOPIH users, habitually used mobile devices and extensively used social media websites and apps. It is logical to think that those who habitually used social media/online platforms would be more likely to use a CoP in a clinical setting because they are already more comfortable with online platforms. Furthermore, the majority of under-50-year-old professionals would have used ICTs intensively at various stages throughout their higher education and professional development. In contrast, many professionals among the over-50-year-old generation could be classified as late adopters of ICTs, mainly because such adoption occurred in the workplace. Consequently, some professionals were reluctant to use ICTs because they saw it as an obligation and considered them hard to use and not particularly useful.

In the explanation of the physicians’ ECOPIH use, second in order of importance was *Perceived usefulness for reducing costs* (time and financial costs). Physicians decided to use the CoCP because they considered that it could become an effective tool for reducing various costs (B = 1.706 *p* = 0.025). Considering time strain, it is reasonable to think that healthcare professionals would opt for the development of more efficient professional activities. Thus, it is understood that the intention to use ECOPIH is conditional upon it being perceived as a tool that enables a correct diagnosis to be made while minimising the amount of time, effort and financial cost involved for both the physicians and the healthcare institutions [9]. However, this tendency appears to be more apparent among younger, especially female professionals than among older male professionals. The setting’s cultural history might explain the gender differences between the two groups identified. Thus, the group of older professionals is mostly male because, at the start of the second half of the twentieth century in Spain, women’s access to certain types of higher education – such as medicine or engineering – was quite limited. The medical profession underwent a gradual feminisation, meaning that those generations of healthcare professionals trained at the end of the last century included a high percentage of women. In the early twenty-first century, 70% of new medical students were women; this has since risen to 85% [48, 49]. Moreover, the younger generation of professionals is very aware of patients’ service experience because of the more active role that patients play in healthcare provision models (empowerment and decision-making) [50] [51].

An important aspect that affects the professionals’ decision to use the tool continuously over time is its ability to fulfil the expectations created in the implementation phase. Regarding the impact of the tool’s use on the number of referrals in an uncontrolled real-life setting, and as seen in other studies [30], the degree of tool use has an influence on the tool’s potential usefulness; this is linked to *Perceived usefulness for reducing costs* (time and financial costs) that ECOPIH use entails. The segment of physicians who predominantly used ECOPIH regularly had lower referral rates in those specialities for which it had been used the most. This particularly reinforces the idea that, for this group of professionals, the tool fulfilled their expectations in terms of its ability to reduce costs associated with clinical practice. These findings are consistent with the results from other studies on the use of telemedicine applied to consultations among professionals [32, 52]. These results indicate that the tool has great potential because we are on the point of a generational changeover. Given today’s user profile, use of ECOPIH, and therefore its usefulness, are expected to increase in the near future.

It should be noted that these results were only obtained in three of the six specialities evaluated (Cardiology, Endocrinology and Gastroenterology, and not Respiratory Medicine, Nephrology or Neurology). This is probably due to two reasons. First, the latter three specialities were incorporated into ECOPIH later, and that might have hindered its use. Second, they are specialities in which fewer referrals are made, probably because the most common disorders within them are more protocolised and less individualised, thereby facilitating an independent handling of them by PC professionals.

Finally, the results obtained show that the professionals in the over-50-year-old profile are those who used ECOPIH to a lesser extent and, in turn, made the most referrals. This segment, as identified in Study 2, bears considerable similarity to Profile 1B observed in Study 1 (except for gender), who used ICTs to a lesser extent and rated information security higher than cost reduction. Hence, it can be seen that the number of referrals made remained unchanged.

### Limitations

This study has a number of limitations. First, the difficulty of recording the impact of such tools should not be overlooked. The impact of CoCP tools should also take into account the quality of referrals, the physician’s trust, and interprofessional communication. Furthermore, use and effectiveness of ECOPIH may have been influenced by other factors not considered in our study. Examples of such factors include: (a) availability of other PC-SC consultation systems; (b) each centre’s healthcare workload, and; (c) being or not being a teaching centre for resident physicians. The results obtained from this study need to be complemented by a qualitative evaluation in order to assess the tool comprehensively [37, 53–55].

Second, we are aware that both the number of ECOPIH users (65 members) and the referral rate (number of referrals per professional) limit the study’s statistical power. In order to solve both of these problems, we could have conducted a randomised controlled study on one specific group of professionals, that is to say, those who were enthusiastic about and committed to using ECOPIH. If we had done so, results of greater magnitude might have been obtained, though it would have affected the external validity of our study, which was conducted in a real-life setting of clinical practice and gave the professionals the freedom to use the tool as they wished, which we believe is one of the greatest strengths of our study.

Finally, during the study design phase, the inclusion of clinical variables as a measure of the effect of ECOPIH use was considered. That option was ultimately rejected owing to the difficulty of isolating the effect of the tool’s use from other influencing factors (e.g., courses taken by the professionals) and of finding a single clinical variable to encapsulate the improved clinical control of patients, since ECOPIH is a platform on which any type of clinical case can be raised for consultation.

### Future implications

We believe that further research should be done on the impact of CoCPs for professionals working in different areas of healthcare to communicate with one another, from the perspective of both the financial implications (a reduction in referrals and visits, and cost analysis) and the clinical outcomes. We propose that longer-term follow-ups should be done and that the use of the tool should be more actively promoted and encouraged, while ensuring that its use is never made compulsory [56]. In order to do that, it will be necessary to ensure that the firm managing the tool guarantees its continuity, that users are given time to actually use it, and that solutions to any technological aspects representing barriers to its use are found. As a future strategy, and in keeping with the recommendations of some authors, it might be appropriate to push ahead with the tool’s dissemination, presenting it in a way that facilitates its use. This would strengthen the available evidence and the relative advantages of using ECOPIH, which would significantly help to increase its use [57].

Finally, it would be interesting to expand the research by looking into the impact of a CoP in a PC clinical setting as a novel tool for training based on real clinical cases.

## Conclusions

ECOPIH and other CoCPs can be used to raise clinical cases for consultation and share information among PC and SC professionals; such tools may reduce the number of referrals to SC. Furthermore, ECOPIH and similar tools offer advantages for clinical efficiency. The potential of the tool increases as more and more young professionals use it. We also believe that its use should be strengthened because of the advantages it offers in terms of efficiency, learning and spreading knowledge.

## Supplementary information

**Additional file 1.** Questionnaire to Primary Care professionals.

**Additional file 2.** Questionnaire to Specialist Care professionals.

## Data Availability

All data generated or analysed during this study are included in this published article.

## Abbreviations

CoCP: Community of clinical practice
CoP: Community of practice
ECOPIH: Eina de Comunicació entre Primària i Hospitalària (Online Communication Tool between Primary and Hospital Care)
ICT: Information and communication technologies
PC: Primary care
PCS: Primary Care Service
SC: Specialist care
UOC: Open University of Catalonia
