# Correction to: Efficiency as a determinant of loyalty among users of a Community of Clinical Practice: a comparative study between the implementation and consolidation phases

**DOI:** 10.1186/s12875-020-01133-w

**Published:** 2020-04-17

**Authors:** David Lacasta Tintorer, Josep Maria Manresa Domínguez, Ana Jiménez-Zarco, Teresa Rodríguez-Blanco, Souhel Flayeh Beneyto, Pere Torán-Monserrat, Xavier Mundet Tuduri, Francesc Saigí-Rubió

**Affiliations:** 1 Centre d’Atenció Primària Gran Sol, Gerència d’Àmbit d’Atenció Primària Metropolitana Nord, Institut Català de la Salut. Avinguda del Doctor Bassols, 112 - 130, 08914 Badalona, Spain; 2 Unitat de Suport a la Recerca Metropolitana Nord, IDIAP Jordi Gol, CAP El Maresme, Camí del Mig, 36 planta 4ª, 08303 Mataró, Spain; 3 Departament de Medicina, Universitat Autònoma de Barcelona, Bellaterra, Cerdanyola del Vallès, Campus de la UAB, Plaça Cívica, s/n, 08193, Bellaterra, Barcelona, Spain; 4 Faculty of Economics and Business, Universitat Oberta de Catalunya, Barcelona, Spain; 5 Institut Universitari d’Investigació en Atenció Primària (IDIAP Jordi Gol), Gran Via Corts Catalanes, 587, àtic, 08007 Barcelona, Spain; 6 Departament de Ciències Mèdiques, Universitat de Girona, C/ Emili Grahit, 77, 2n, 17003 Girona, Spain; 7 Unitat de Suport a la Recerca Barcelona Ciutat, IDIAP Jordi Gol, Carrer Sardenya 375, 08025 Barcelona, Spain; 8 Faculty of Health Sciences, Universitat Oberta de Catalunya, Barcelona. Av. Tibidabo, 39-43, 08035 Barcelona, Spain

**Correction to: BMC Fam Pract (2020) 21:15**

**https://doi.org/10.1186/s12875-020-1081-x**

Following publication of the original article [1], in order to comply with the current regulations for the submission of Doctoral Thesis by compendium of articles, the Universitat Autónoma de Barcelona Doctoral School asks us to update the affiliation number 3, adding “Departament de Medicina” at the beginning, as follows:

3 Departament de Medicina Universitat Autònoma de Barcelona, Bellaterra, Cerdanyola del Vallès. Campus de la UAB, Plaça Cívica, s/n, 08193 Bellaterra, Barcelona, Spain.
