# Supplementary material for: Efficiency as a determinant of loyalty among users of a Community of Clinical Practice: a comparative study between the implementation and consolidation phases
Source: BMC Fam Pract. 2020 Jan 24;21:15. doi: 10.1186/s12875-020-1081-x (PMC6979059; doi:10.1186/s12875-020-1081-x)
Supplement: Supplementary file 1 — Additional file 1. Questionnaire to Primary Care professionals. [file 12875_2020_1081_MOESM1_ESM.pdf]

ECOPIH SURVEY

(initial text – presentation)

Note: questionnaire to Primary Care professionals.

PERSONAL INFORMATION

1. What is your year of birth? ☐

2. What is your sex?

Male	☒
Female	☒

3. What is your professional category?

Doctor	☒
Nurse	☒

4. Does your health centre have any face-to-face consultancies with the following specialities?

	Yes	No
Cardiology	☒	☒
Pneumology	☒	☒
Endocrinology	☒	☒
Any others (please specify)		

5. On a scale of 1 to 10, where 1 is "NOT AT ALL" and 10 is "COMPLETELY", how much do you agree with the following statements about organizational aspects of your health centre?

	1	2	3	4	5	6	7	8	9	10
The organization of work and pay at your health centre is target-related.	☒	☒	☒	☒	☒	☒	☒	☒	☒	☒
Staff have the freedom to organize their own work.	☒	☒	☒	☒	☒	☒	☒	☒	☒	☒
Staff can propose targets.	☒	☒	☒	☒	☒	☒	☒	☒	☒	☒
Teamwork is promoted.	☒	☒	☒	☒	☒	☒	☒	☒	☒	☒
Staff's multi-tasking is valued.	☒	☒	☒	☒	☒	☒	☒	☒	☒	☒
Communication between care levels (primary care – specialist) needs to be improved.	☒	☒	☒	☒	☒	☒	☒	☒	☒	☒

LEVEL OF USE OF DIGITAL TECHNOLOGIES AND INTERNET IN GENERAL

6. Which of the following devices do you normally use?

	Care provision	Research	Personal use	I do not use it
Smartphone	☒	☒	☒	☒
Desktop computer	☒	☒	☒	☒
Laptop	☒	☒	☒	☒
Tablet	☒	☒	☒	☒
Handheld PC (PDA, Palm)	☒	☒	☒	☒
iPad (or similar)	☒	☒	☒	☒

7. Which of these platforms do you use regularly (at least once a week)?

	Care provision	Research	Personal use	I do not use it
Facebook	☒	☒	☒	☒
Twitter	☒	☒	☒	☒
Google+	☒	☒	☒	☒
Personal blog	☒	☒	☒	☒
Other people's blogs	☒	☒	☒	☒
LinkedIn	☒	☒	☒	☒
Other (please specify)				

USE OF ECOPIH

8. Are you registered with ECOPIH?

Yes ☒

No ☒

If you aren't, please go on to question 15.

9. Do you think that the training you've received about how the ECOPIH tool works and its possibilities is sufficient?

Yes	☐
No	☐
Don't know / prefer not to say	☐

10. How often do you do the following tasks on ECOPIH?

	Daily	Weekly	Monthly	Occasionally	Never
Read content.	☐	☐	☐	☐	☐
Raise queries with the specialist.	☐	☐	☐	☐	☐
Make a contribution (blog post, comment, referrals, etc).	☐	☐	☐	☐	☐
Take part in debates about a query.	☐	☐	☐	☐	☐
Upload documents (studies, guides, etc).	☐	☐	☐	☐	☐

11. What do you think ECOPIH is useful for?

Training	☐
Care	☐
Both	☐
Neither	☐

12. Please state how much you agree with the following statements:

	Disagree				Agree completely
The e-Catalunya platform that ECOPIH uses is relatively user-friendly.	☐	☐	☐	☐	☐
The e-Catalunya platform that ECOPIH uses displays information relatively well.	☐	☐	☐	☐	☐
The time it takes for my questions to be answered on ECOPIH fulfils my needs.	☐	☐	☐	☐	☐
The content on ECOPIH is good quality.	☐	☐	☐	☐	☐
It is easy to consult specialists about cases.	☐	☐	☐	☐	☐
It is useful being able to consult past cases to help resolve current problems.	☐	☐	☐	☐	☐
ECOPIH enables patient confidentiality.	☐	☐	☐	☐	☐
ECOPIH enables reductions in the number of referrals.	☐	☐	☐	☐	☐
ECOPIH enables improvements in the quality of the referrals.	☐	☐	☐	☐	☐
ECOPIH enables improvements in the care patients receive.	☐	☐	☐	☐	☐
ECOPIH improves communication between care levels.	☐	☐	☐	☐	☐

13. Is there any speciality currently not on ECOPIH that you feel it would be useful to add?

Yes ☐

No ☐

Don't know / prefer not to say ☐

14. ***If you answered yes to the previous question,*** **which speciality?**

Please state which speciality you feel feel it would be useful for ECOPIH to add.

15. Do you have any comments or suggestions for improvements?
