# Supplementary material for: Efficiency as a determinant of loyalty among users of a Community of Clinical Practice: a comparative study between the implementation and consolidation phases
Source: BMC Fam Pract. 2020 Jan 24;21:15. doi: 10.1186/s12875-020-1081-x (PMC6979059; doi:10.1186/s12875-020-1081-x)
Supplement: Supplementary file 2 — Additional file 2. Questionnaire to Specialist Care professionals. [file 12875_2020_1081_MOESM2_ESM.pdf]

ECOPIH SURVEY

(initial text – presentation)

Note: questionnaire to Specialist Care profesionals

PERSONAL INFORMATION

1. What is your year of birth?

2. What is your Sex?

Male ☐

Female ☐

3. What is your professional category?

Specialist physician ☐

Nurse ☐

Psychologist ☐

Physiotherapist ☐

Occupational therapist ☐

Other

4. In what area do you specialize?

Specialization

5. Does your centre provide consultancy services to any primary healthcare providers?

Yes, face-to-face ☐

Yes, online (email, videoconferencing, etc) ☐

No ☐

Don’t know / Prefer not to say ☐

6. On a scale of 1 to 10, where 1 is “NOT AT ALL” and 10 is “COMPLETELY”, how much do you agree with the following statements about organizational aspects of your health centre?

	1	2	3	4	5	6	7	8	9	10
The organization of work and pay at your health centre is target-related.	☐	☐	☐	☐	☐	☐	☐	☐	☐	☐
Staff have the freedom to organize their own work.	☐	☐	☐	☐	☐	☐	☐	☐	☐	☐
Staff can propose targets.	☐	☐	☐	☐	☐	☐	☐	☐	☐	☐

Teamwork is encouraged.	☒	☒	☒	☒	☒	☒	☒	☒	☒	☒
Staff's multi-tasking is valued.	☒	☒	☒	☒	☒	☒	☒	☒	☒	☒
Communication between care levels (primary care – specialist) needs to be improved.	☒	☒	☒	☒	☒	☒	☒	☒	☒	☒

LEVEL OF USE OF DIGITAL TECHNOLOGIES AND INTERNET IN GENERAL

7. Which of the following devices do you normally use?

	Care provision	Research	Personal use	I do not use it
Smartphone	☒	☒	☒	☒
Desktop computer	☒	☒	☒	☒
Laptop	☒	☒	☒	☒
Tablet	☒	☒	☒	☒
Handheld PC (PDA, Palm)	☒	☒	☒	☒
iPad (or similar)	☒	☒	☒	☒

8. Which of these platforms do you use regularly (at least once a week)?

	Care provision	Research	Personal use	I do not use it
Facebook	☒	☒	☒	☒
Twitter	☒	☒	☒	☒
Google+	☒	☒	☒	☒
Personal blog	☒	☒	☒	☒
Other people's blogs	☒	☒	☒	☒
LinkedIn	☒	☒	☒	☒
Others (please specify)				

USE OF ECOPIH

9. Are you registered with ECOPIH?

Yes	☒
No	☒

If you aren't, please go on to question 15.

10. Do you think that the training you've received about how the ECOPIH tool works and its possibilities is sufficient?

Yes	☒
No	☒
Don't know / prefer not to say	☒

11. How often do you do the following things on ECOPIH?

	Daily	Weekly	Monthly	Occasionally	Never
Answer primary care providers' questions.	☒	☒	☒	☒	☒
Read content.	☒	☒	☒	☒	☒
Make a contribution (blog post, comment, etc).	☒	☒	☒	☒	☒
Take part in debates about a query.	☒	☒	☒	☒	☒
Upload documents (studies, guides, etc).	☒	☒	☒	☒	☒

12. What do you think ECOPIH is useful for?

Training	☒
Care	☒
Both	☒
Neither	☒

13. Please state how much you agree with the following statements:

	Disagree				Agree completely
The e-Catalunya platform that ECOPIH uses is relatively user-friendly.					
The e-Catalunya platform that ECOPIH uses displays information relatively well.					
The content on ECOPIH is good quality.					
It is easy to read the cases for which consultations are made.					
It is useful being able to consult past cases to help resolve current problems.					
ECOPIH enables patient confidentiality.					
ECOPIH enables reductions in the number of referrals.					
ECOPIH enables improvements in the quality of the referrals.					
ECOPIH enables improvements in the care patients receive.					
ECOPIH improves communication between care levels.					

14. Do you have any comments or suggestions for improvements?
